# Fungal Seed Endophyte FZT214 Improves *Dysphania ambrosioides* Cd Tolerance Throughout Different Developmental Stages

**DOI:** 10.3389/fmicb.2021.783475

**Published:** 2022-01-04

**Authors:** Shobhika Parmar, Vijay K. Sharma, Tao Li, Wenting Tang, Haiyan Li

**Affiliations:** ^1^Medical School of Kunming University of Science and Technology, Kunming, China; ^2^State Key Laboratory for Conservation and Utilization of Bio-resources in Yunnan, Yunnan University, Kunming, China

**Keywords:** seed endophytes, heavy metal tolerance, plant developmental stages, synthetic seeds, plant microbe interactions, fungal endophyte

## Abstract

Phytoremediation is a promising remediation method of heavy metal (HM)–contaminated soils. However, lower HM tolerance of metal accumulator inhibits its practical application and effects. The current study was aimed to illustrate the role of fungal seed endophyte (FZT214) in improving *Dysphania ambrosioides* Cd tolerance during different developmental stages under various Cd stresses (5, 15, 30 mg kg^–1^) by pot experiments. The results showed that FZT214 significantly (*p* < 0.05) improved the host plant’s growth at the flowering and fruiting stage in most of the treatment, while at the growing stage the increase was less (*p* > 0.05). The seed yield was also improved (*p* < 0.05) in the FZT214-inoculated plants (E+) and induced early flowering was observed. Moreover, the inoculation also positively affected total chlorophyll content, antioxidant process, and lipid peroxidation in most of the treatments throughout three developmental stages. Not all but in most cases, IAA and GA were more in E+ plants while JA was more in the E− plants (non-inoculated plants) during three developmental stages. The results suggested that the colonization of FZT214 to the *D. ambrosioides* might trigger multiple and comprehensive protective strategies against Cd stress, which mainly include activation of the dilution effects, induced biochemical changes to overcome damage from Cd toxicity, and alteration of the endogenous phytohormones. FZT214 can find competent application in the future to improve the growth of other crop plants.

## Introduction

The existence of potentially toxic elements (PTEs) in the surrounding environment causes numerous ecological consequences (Chen et al., 2014). These elements often persist in the environment for a long period due to their non-degradable nature and are difficult to be broken into less toxic forms. Cadmium (Cd) is recognized as a PTE that is transferred into the surrounding ecosystem either naturally or through anthropogenic activities such as discharge of industrial wastes, mining wastes, smelting, uses of sewage sludge for agricultural purpose, etc. (Gallego et al., 2012; Robson et al., 2014). Yunnan Province, China, is full of valuable metal resources such as lead (Pb), zinc (Zn), Cd, and copper (Cu) (Yanqun et al., 2004), and some of these metal mines are as big as 26.053 million tons (Bai et al., 1985) and mining has been carried out for more than 300 years (Parmar et al., 2018). Cd is a non-biodegradable, highly toxic, and persistent metal pollutant, which causes numerous diseases, for example, kidney disorders, neurotoxicity, and osteoporosis (Park et al., 2012). Although Cd is a non-essential element that causes toxicity in crop plants (Kuriakose and Prasad, 2008), it can be translocated to vegetative parts including seeds (Sharma et al., 2006). Cd has great mobility and water solubility; its transport to the growing plant from the contaminated soil depends on bioavailability and several cation transporter elements such as calcium (Ca), iron (Fe), and Zn (Aravind and Prasad, 2005; Chirila and Carazeanu, 2008; He et al., 2019). Subsequently, from the contaminated plants, Cd enters the food chain and induces toxic symptoms on all living organisms and human beings. Besides, it is carcinogenic to humans even at low concentrations (Khan et al., 2015). Chlorosis of leaf, necrosis of leaf and root, reduced growth, genomic DNA damage, initiation of cell death, photosynthetic inhibition, oxidative stress, and lipid membrane damage are some of the major symptoms of Cd toxicity (Hernandez and Cooke, 1997; Kabata-Pendias and Pendias, 2001; Clemens, 2006; Groppa et al., 2007; Xu et al., 2013; Lomaglio et al., 2015; Rizwan et al., 2016). Cd was listed at the seventh position for the observed toxicity by the Agency for Toxic Substances and Disease Registry in 1997 (Liao and Freedman, 1998). According to the “National Soil Pollution Survey Bulletin” release in 2014 by the Chinese Ministry, the extreme level of Cd reached 7.0% (Liu et al., 2019). Therefore, the remediation of Cd-contaminated soil is of utmost importance to maintain the ecological balance and provide safe food to mankind.

For the remediation of the PTEs in the soil, several physical and chemical methods can be employed like soil flushing and stabilization by a suitable sorbent etc., but these methods come with some limitations such as high cost and labor (Laghlimi et al., 2015; Parmar and Singh, 2015). Phytoremediation using metal accumulators is one of the low-cost and environment-friendly methods for the extraction or stabilization of toxic metals from contaminated soils. Metal phytotoxicity is evident and well known in the contaminated soils, but often plants growing in contaminated soil having a long history of PTEs become tolerant and accumulate high amounts of PTEs. But how these plants can survive excessive PTEs is a matter of investigation and scientific importance.

*Dysphania ambrosioides* (L.) is an invasive plant in China that was also known as *Chenopodium ambrosioides* in the past and reported as a Pb hyperaccumulator (Wu et al., 2004). It is a dominant plant in the Pb–Zn contaminated mining areas in Huize County, Yunnan Province, Southwest China (Li et al., 2012, 2016). Our previous studies have revealed that *D. ambrosioides* growing in Pb–Zn contaminated locations were colonized with a high diversity of bacterial and fungal endophytes (Li et al., 2016; Parmar et al., 2018; Sun et al., 2019). Some of these endophytes demonstrated Pb, Zn, and Cd tolerance properties and enhanced host plant growth and influenced its metal accumulation (Li et al., 2016; Sun et al., 2019). The possible mechanism of the endophyte-induced stress tolerance to the host plant growing in soil containing high amounts of metal involves metal detoxification, altering metal distribution in plant cells and positively affecting the antioxidative system (Wang et al., 2016). Several recent studies have reported that the plant-associated symbiotic microbes can affect translocation and accumulation of Cd, improve Cd tolerance, and can enhance the host plant growth under Cd stress conditions (Wang et al., 2016, 2019; Zhang et al., 2019; Li et al., 2020; Yung et al., 2021). Apart from the microbial assemblage associated with the foliar and belowground plant tissues, a portion of the microbial community is conserved into the seeds for the next generation (Walitang et al., 2019). Vertically transmitted seed endophytes are the first symbiotic microbes that colonize the young seedling and subsequently determine the fate of the plant (Li et al., 2019). The high association with the seed endophytes and their potential functional role in increasing host plant tolerance against abiotic stress, such as salt and metal stress (Walitang et al., 2018; Li et al., 2019), make these endophytes the most suitable candidate for the microbial-engineered plant with improved beneficial traits. For example, growth-promoting characteristics conferred by a seed endophyte will be automatically transferred to the subsequent plant generation through the seeds.

In our previous studies, the fungal endophyte FZT214 isolated from the seeds of *D. ambrosioides* showed better Pb, Zn, and Cd tolerance, and can improve host plants’ seed germination and seedling growth under Cd stress. However, the mechanism is unknown. Thus, the present study was designed to understand the way seed endophyte FZT214 improves host plant (*D. ambrosioides*) Cd tolerance at different developmental stages, which includes host plant growth promotion, important stress-related biochemical factors, and endogenous changes to the phytohormones levels.

## Materials and Methods

### The Fungal Seed Endophyte FZT214

The fungal endophyte FZT214 was isolated from the seeds of *D. ambrosioides* and was identified to be *Epicoccum nigrum* based on morphology characteristics and molecular analysis (GenBank accession number MN847628.1).

### Pot Experiments

For the pot experiments, the seeds of *D. ambrosioides* were surface sterilized by sequentially dipping in 75% ethanol for 2 min, followed by 5% sodium hypochlorite for 2 min and finally 3–5 times rinsed with sterile distilled water (Li et al., 2012). The isolate FZT214 was cultured on PDA plates at 25°C for 4–7 days, and the mycelia were collected and cut into small fragments and suspended in autoclaved distilled water to obtain the mycelia suspension. Then, the surface-sterilized seeds were imbibed in the mycelia suspension for 1 h at 28°C. The control seeds were soaked into an equal volume of autoclaved mycelia suspension and kept under the same conditions. Subsequently, these seeds were germinated on sterile-water-moistened filter paper in autoclaved Petri dishes under aseptic conditions in a growth chamber (26 ± 1 / 18 ± 1°C, 16/8 h light/dark cycle, 60% relative humidity) for 21 days. The germinated seedlings, equal in size, were transferred to plastic pots (three seedlings per pot) containing sterilized soil substrate (30% perlite:70% peat moss, vol/vol). The soil substrate was supplied with the overages of CdCl_2_⋅2.5H_2_O to the final concentration of 0, 5, 15, and 30 mg kg^–1^ Cd. The pots were arranged randomly and kept under artificial plant light (16:8 h light/dark cycle). The plants were watered with autoclaved water every 2–3 days according to the requirement and supplied with a nutrient solution once a week. The inoculated plants (E+) were sprayed with the mycelia suspension of FZT214 at 30, 60, 90, and 120 days of the transplant while the control/non-inoculated plants (E−) were sprayed with the autoclaved mycelia suspension of FZT214 in equal amount. There were eight treatments: four Cd concentrations in combination with E+ and E− plants and three developmental stages. Each treatment involved three replicate pots and each pot has three plants. In addition, for each treatment, two pots were kept to recover mature seeds. Thus, the full experiment consists of 88 (72+16) pots.

### Plant Growth Attributes and Cd Content Analysis

The plants were harvested at 40 days (growing stage or pre-flowering stage), 105 days (flowering stage), and 130 days (fruiting stage). After harvest, the fresh leaves were collected from each treatment plant, immediately frozen in liquid nitrogen, stored at −80°C, and used for biochemical and phytohormone analysis within 2 weeks. The plants were separated into the shoot (all aboveground parts) and roots (all belowground parts) and washed under tap water to remove adhered soil particles, and then rinsed with deionized water. Finally, the shoot and root lengths were measured. Thereafter, the plants were put into paper bags and oven dried at 50–60°C to constant weight, then the dry biomass and Cd concentration were tested. The rhizospheric soil from each treatment was collected, air dried, and used for Cd analysis. Two pots of each treatment were kept under the same growing conditions for seed maturation. Approximately after 6 months, the seeds were collected, and dry weight was recorded.

For the Cd content analysis, the dried plant samples were crushed to fine powders with a mortar and pestle, and 0.2 g roots/shoots powders was digested with 5 ml HNO_3_ (65% w/w) at 110°C for 2 h, then cooled and added with 1 ml H_2_O_2_ (30% w/w) and heated for 1 h. Finally, the digests were diluted to 50 ml with triple deionized water in a volumetric flask (Shen et al., 2013). The total Cd concentration in the soil was determined by digesting 0.5 g fine soil powder with 4 ml HCl–HNO_3_ (3:1, v/v) mixture at 80°C for 30 min, then 100°C for 30 min, and finally 120°C for 1 h. Thereafter, cooled and 1 ml HClO_4_ was added to continue digestion at 100°C for 20 min, followed by 120°C for 1 h. Finally, the digests were diluted to 50 ml with triple deionized water in a volumetric flask. The concentrations of bio-available Cd in soils were extracted by diethylenetriaminepentaacetic acid–triethanolamine (DTPA-TEA) (Huang et al., 2006). All the samples were prepared in triplicates. The concentrations of Cd in plant parts and soil digests were determined by flame atomic absorption spectrometry (Li et al., 2014). The mean and SD of the HM concentrations were calculated using triplicates, with each replicate consisting of mixed plant parts and soil samples from the individual pot.

### Estimation of Total Chlorophyll

Just before the harvesting of the plants at each growth stage, the total chlorophyll was measured with a chlorophyll meter (SPAD 502 plus; Konica Minolta, Inc., Tokyo, Japan). Each individual recorded value of the chlorophyll was average of the 10 readings from the youngest fully developed leaves of the same plant.

### Bioaccumulation and Translocation Analysis

Bioaccumulation factor (BAF) and translocation factor (TF) are used in the monitoring of plant’s phytoremediation efficiency (Testiati et al., 2013). To assess Cd translocation ability from root to shoot of plants, TF was estimated as per Khan et al. (2015).


$$\text{TF}=\frac{\begin{array}{c} \mathrm{Metal}\,\mathrm{concentration}\,\mathrm{in}\,\mathrm{the}\\ \mathrm{aboveground}\,\mathrm{plant}\,\mathrm{parts}\,\left(\mathrm{mg}\,{\mathrm{kg}}^{-1}\right) \end{array}}{\begin{array}{c} \mathrm{Metal}\,\mathrm{concentration}\,\mathrm{in}\,\mathrm{the}\\ \mathrm{belowground}\,\mathrm{plant}\,\mathrm{parts}\,\left(\mathrm{mg}\,{\mathrm{kg}}^{-1}\right) \end{array}}$$

BAF was calculated as the ratio of Cd accumulation in the shoots to the Cd accumulation in the soil. In short, the BAF explains the potential of plants to absorb metal from the soil and subsequently and translocate it to aboveground tissues. The BAF was estimated as defined by Sharma et al. (2019).


$$\text{BAF}=\frac{\begin{array}{c} \mathrm{Metal}\,\mathrm{concentration}\,\mathrm{in}\,\mathrm{the}\\ \mathrm{aboveground}\,\mathrm{plant}\,\left(\mathrm{mg}\,{\mathrm{kg}}^{-1}\right) \end{array}}{\begin{array}{c} \mathrm{Metal}\,\mathrm{concentration}\,\mathrm{in}\,\mathrm{the}\\ \text{soil}\,\left(\mathrm{mg}\,{\mathrm{kg}}^{-1}\right) \end{array}}$$

### Lipid Peroxidation Analysis

The lipid peroxidation was measured by evaluating the malondialdehyde (MDA) using commercial chemical assay kits (Nanjing Jiancheng Bioengineering Institute, China) according to the manufacturer’s protocol. The test was performed in triplicates. The extent of lipid peroxidation was expressed in terms of nanomoles of malondialdehyde (MDA) formation per gram of leaf tissue. Briefly, the frozen leaf tissue sample was homogenized with a mortar and pestle in a chilled phosphate buffer (50 mM, pH 7.2). The tissue homogenate was centrifuged at 3,500 rpm for 10 min at 4°C. After centrifugation, the resulting supernatant was collected and used to measure MDA concentration using a spectrophotometer (MAPADA UV-1800 PC).

### Determination of Glutathione

Glutathione a tripeptide compound having important functions in the antioxidant processes involved in the plant defense mechanism and also a metal chelator (Guo et al., 2012). The reduced glutathione (GSH) was determined using the total glutathione (T-GSH) and oxidized glutathione (GSSG) assay kit (Nanjing Jiancheng Bioengineering Institute, Nanjing, China). The methods were principally based on the previously described enzymatic recycling method evolving cyclic reaction of DTNB (Rahman et al., 2006). Briefly, frozen leaves were homogenized in extraction buffer (1:4 ratio, wt/vol). Afterward, the homogenate was centrifuged at 3,500 rpm for 10 min at 4°C, the supernatant was collected, and stored at 4°C (−20°C if required to keep overnight) until further analysis according to the manufacturer’s protocol. The absorbance of the assay mixture was measured twice at 412 nm, first at 30 s and second at 10 min 30 s of the reaction initiation. T-GSH and GSSG content were determined using given formulas. The GSH content was calculated by subtracting the GSSG content from the T-GSH content according to the formula provided in the kit, expressed in micromoles per gram fresh leaf weight.

### Determination of the Plant Hormones

Frozen leaves (about 200 mg fresh weight) were crushed in liquid nitrogen with a mortar and pestle then extracted with 10 ml of chilled ethanol. The extracts were vortexed. Subsequently, the extracts were centrifuged at 4,000 rpm for 15 min at 4°C. The supernatant was collected and used for the determination of jasmonic acid (JA), gibberellic acid (GA), and indole-3-acetic acid (IAA). The ELISA-based phytohormone kits from MLBIO Biotechnology Co., Ltd., Shanghai^1^ were used for the assay according to the manufacturer’s instructions. The color change of the reaction mixture was measured spectrophotometrically at a wavelength of 450 nm. The concentrations of JA, GA, and IAA in the extracts were determined by comparing the O.D. of the samples to the standard curve plotted with the provided standards. The test was performed in triplicate; each replicate consisted of the leaves of all three plants of a single pot pooled together as one sample.

To determine IAA, GA, and JA in crude secondary metabolites of FZT214, the endophytic isolate was cultured in potato dextrose broth for 21 days at 28 ± 2°C in a BOD incubator. Thereafter, the broth culture was filtered and extracted thrice in equal volume ethyl acetate. The extracted metabolites were concentrated in a vacuum rotary evaporator and dissolved in methanol. Subsequently, this crude secondary metabolite of FZT214 was tested for phytohormones as described previously.

### Statistical Analysis

The results are presented as the mean of the replicates and SD for each treatment, calculated using Excel 2007. One-way ANOVA, Duncan test (*p* < 0.05) was performed using SPSS 16.0 to determine the differences in mean values between the different treatments of inoculated and non-inoculated plants, and *p-*value was set at < 0.05. The figures were generated through Origin V8.0724 software.

## Results

### Effects of FZT214 on the Growth of *D. ambrosioides*

**FIGURE 1:**
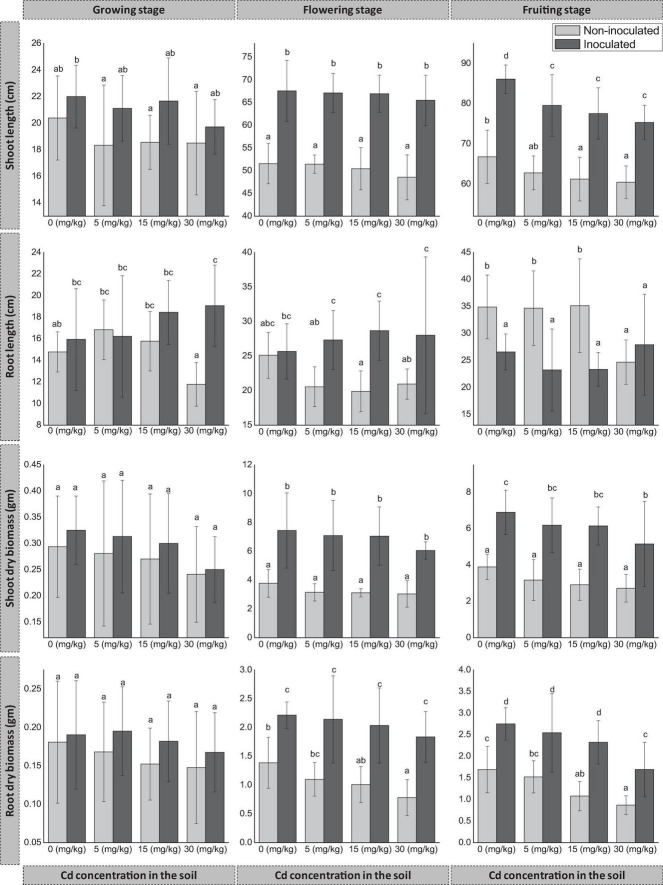
Effect of seed endophyte FZT214 on *D. ambrosioides* growth under different Cd concentrations at different developmental stages (growing, flowering, and fruiting). The different lowercase letters indicate significant variation between treatments (*p* < 0.05) according to Duncan’s test of one-way ANOVA. The shoot and root length, dry weight (mean ± STD, *n* = 9).

Pot experiments indicated that seed endophyte FZT214 supported the host plant’s growth throughout different developmental stages under variable Cd stress (Figure 1).

#### Aboveground Growth

The shoot length significantly improved (*p* < 0.05) at the flowering and fruiting stage, while at the growing stage there was an increase but not significant (*p* > 0.05). However, the shoot length decreased with the increase in Cd concentration in the amended soil substrate for both inoculated (E+) and non-inoculated plants (E−) (Figure 1). At all three stages, the dry biomass of E+ plant shoot was more than that of E− plant shoot irrespective of the Cd concentrations. The difference was significant (*p* < 0.05) at the flowering and fruiting stages, while it was non-significant (*p* > 0.05) at the growing stage (Figure 1).

#### Belowground Growth

During the growing stage, the root length of E+ plants was more than that of E− plants at all Cd concentrations except 5 mg kg^–1^. The difference was significant (*p* < 0.05) at 30 mg kg^–1^ Cd. Also during the flowering stage, the root length of E+ plants was more than that of E− plants at all Cd concentrations; the difference was significant (*p* < 0.05) at 5, 15, and 30 mg kg^–1^ Cd while non-significant (*p* > 0.05) at 0 mg kg^–1^ Cd. However, during the fruiting stage, the root length of E− plants was more than that of E+ plants at all Cd concentrations except at 30 mg kg^–1^, and the difference was significant (*p* < 0.05) except at 30 mg kg^–1^. The dry biomass of E+ plant root was more than that of E- plants at different developmental stages and Cd concentrations. The difference was significant (*p* < 0.05) at the flowering and fruiting stages, while it was non-significant (*p* > 0.05) at the growing stage.

#### Seed Production

**FIGURE 2:**
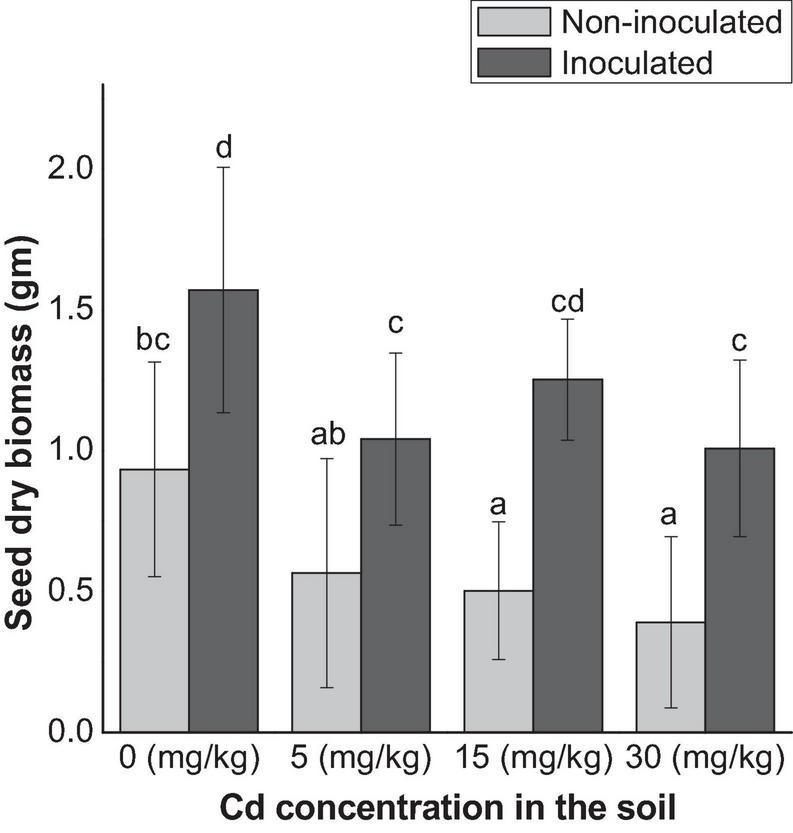
Seed production affected by endophyte FZT214 under variable Cd stress. The different lowercase letters indicate significant variation between treatments (*p* < 0.05) according to Duncan’s test of one-way ANOVA (mean ± STD, *n* = 6).

The seed production decreased both in E+ and E− plants as the Cd increase in the soil, except at 15 mg kg^–1^ Cd for E+ plants. Overall, seed production was significantly (*p* < 0.05) higher in E+ plants than that of E− plants (Figure 2).

### Effects of FZT214 on Cd Accumulation, Uptake, and Translocation

Cd uptake and accumulation in aboveground and belowground parts of E+ and E− plants at different Cd concentrations are presented in Table 1. It was found that Cd concentrations decreased in aboveground parts of E+ plants as compared with E− plants except at 30 mg kg^–1^ Cd at the growing stage and 5 mg kg^–1^ Cd at the fruiting stage. Similarly, Cd concentrations also decreased in belowground parts of E+ plants as compared with E− plants except at 5 mg kg^–1^ Cd at the growing stage. However, the difference was non-significant (*p* > 0.05) in most of the tested conditions except for Cd accumulation in aboveground parts at 15 mg kg^–1^ Cd during the flowering and fruiting stage, and at 30 mg kg^–1^ Cd during the growing stage.

**TABLE 1:** Effects of endophyte FZT214 on Cd accumulation in aboveground and belowground tissues of *D. ambrosioides* grown under different Cd concentrations at different developmental stages (mean ± STD, *n* = 3).

		0 mg Cd kg^–1^ soil		5 mg Cd kg^–1^ soil		15 mg Cd kg^–1^ soil		30 mg Cd kg^–1^ soil	
		Non-inoculated	Inoculated	Non-inoculated	Inoculated	Non-inoculated	Inoculated	Non-inoculated	Inoculated
**Tissue cadmium content (mg kg^–1^ dry weight)**									
Aboveground parts	Growing stage	0.30 ± 0.05a	0.30 ± 0.03a	12.30 ± 1.71b	12.07 ± 1.63b	21.31 ± 3.52cd	17.62 ± 4.04c	23.63 ± 3.31d	28.80 ± 0.48e
	Flowering stage	0.38 ± 0.12a	0.27 ± 0.02a	6.41 ± 0.77b	4.95 ± 0.89b	11.13 ± 1.94d	8.33 ± 0.51c	11.13 ± 1.45d	9.21 ± 1.54cd
	Fruiting stage	0.20 ± 0.02a	0.13 ± 0.00a	5.47 ± 1.50b	5.83 ± 1.16b	9.82 ± 2.27c	6.56 ± 1.10b	10.69 ± 1.58c	10.27 ± 0.60c
Belowground parts	Growing stage	0.59 ± 0.13a	0.26 ± 0.03a	15.48 ± 2.18ab	19.40 ± 5.68b	22.70 ± 6.60b	18.68 ± 4.25b	47.74 ± 23.29c	39.05 ± 7.86c
	Flowering stage	0.78 ± 0.32a	0.31 ± 0.04a	17.74 ± 2.31bc	13.63 ± 3.62b	17.45 ± 1.59bc	17.15 ± 2.06bc	20.85 ± 1.96c	20.30 ± 7.46c
	Fruiting stage	0.56 ± 0.10a	0.45 ± 0.18a	19.44 ± 2.27bc	11.75 ± 1.08b	22.79 ± 5.54bc	13.53 ± 2.19bc	20.45 ± 0.71c	17.25 ± 5.95c

*The different lowercase letters indicate significant variation between treatments (p<0.05) according to Duncan’s test of one-way ANOVA.*

The total and the bioavailable Cd content of the rhizospheric soil are presented in Table 2. In general, the total and bioavailable Cd in the rhizospheric soil of E+ plants was less than that of E− plants, except for 5 and 15 mg kg^–1^ Cd in the growing and flowering stages. The BAF and TF are presented in Table 3. The results indicated that the inoculation of FZT214 to *D. ambrosioides* did not significantly (*p* > 0.05) affect the BAF and TF for Cd, with only a few exceptions.

**TABLE 2:** Effects of endophyte FZT214 on total and bioavailable Cd in the soils at different developmental stages.

	0 mg Cd kg^–1^ soil		5 mg Cd kg^–1^ soil		15 mg Cd kg^–1^ soil		30 mg Cd kg^–1^ soil	
	Non-inoculated	Inoculated	Non-inoculated	Inoculated	Non-inoculated	Inoculated	Non-inoculated	Inoculated
**Total Cd content of the rhizospheric soil after harvest**								
Growing stage	0.36 ± 0.16a	0.20 ± 0.065a	7.64 ± 0.85b	10.31 ± 2.10b	36.78 ± 2.30d	29.42 ± 0.79c	96.27 ± 2.09f	70.23 ± 7.12e
Flowering stage	0.47 ± 0.12a	0.36 ± 0.04a	35.63 ± 2.66c	22.52 ± 3.71b	44.33 ± 7.93d	60.53 ± 6.84e	66.66 ± 3.93e	63.78 ± 7.08e
Fruiting stage	0.63 ± 0.03a	0.53 ± 0.05a	37.13 ± 7.88b	27.30 ± 2.20b	67.09 ± 3.69c	63.53 ± 5.45c	85.65 ± 8.29d	62.68 ± 12.20c
**Bioavailable Cd content of the rhizospheric soil after harvest**								
Growing stage	0.29 ± 0.14a	0.18 ± 0.06a	5.36 ± 0.55b	6.98 ± 1.31b	27.11 ± 1.17d	20.36 ± 0.53c	71.90 ± 1.05f	54.32 ± 5.14e
Flowering stage	0.37 ± 0.14a	0.26 ± 0.04a	24.38 ± 1.90c	15.14 ± 2.60b	31.16 ± 5.58c	45.57 ± 5.21d	51.08 ± 1.72d	48.17 ± 8.79d
Fruiting stage	0.54 ± 0.03a	0.43 ± 0.05a	27.91 ± 6.75c	17.76 ± 2.00b	53.70 ± 5.47d	45.78 ± 4.61d	73.92 ± 1.34e	47.07 ± 9.82d

*The different lowercase letters indicate significant variation between treatments (p < 0.05) according to Duncan’s test of one-way ANOVA.*

**TABLE 3:** Effects of the endophyte (FZT214) inoculation on bioaccumulation factor (BAF) and translocation factor (TF) (mean ± STD, n = 3) of *D. ambrosioides* grown under different Cd concentrations at different developmental stages (growing, flowering, and fruiting).

		5 mg Cd kg^–1^ soil		15 mg Cd kg^–1^ soil		30 mg Cd kg^–1^ soil	
		Non-inoculated	Inoculated	Non-inoculated	Inoculated	Non-inoculated	Inoculated
BAF	Growing stage	1.61 ± 0.08d	1.20 ± 0.29c	0.58 ± 0.13b	0.60 ± 0.15b	0.25 ± 0.03a	0.41 ± 0.04ab
	Flowering stage	0.18 ± 0.02ab	0.23 ± 0.07ab	0.26 ± 0.09b	0.14 ± 0.02a	0.17 ± 0.03ab	0.15 ± 0.04a
	Fruiting stage	0.15 ± 0.01ab	0.21 ± 0.05c	0.15 ± 0.03ab	0.10 ± 0.01a	0.12 ± 0.01ab	0.17 ± 0.03bc
TF	Growing stage	0.82 ± 0.22abc	0.64 ± 0.10ab	0.97 ± 0.22c	0.94 ± 0.06bc	0.55 ± 0.16a	0.76 ± 0.13abc
	Flowering stage	0.36 ± 0.02a	0.37 ± 0.04a	0.63 ± 0.06b	0.49 ± 0.08ab	0.54 ± 0.10ab	0.51 ± 0.23ab
	Fruiting stage	0.29 ± 0.11a	0.50 ± 0.11ab	0.44 ± 0.08ab	0.49 ± 0.11ab	0.52 ± 0.09ab	0.65 ± 0.24b

*The different lowercase letters indicate significant variation between treatments (p < 0.05) according to Duncan’s test of one-way ANOVA.*

### Effects of FZT214 on the Biochemical Factors of *D. ambrosioides*

#### Chlorophyll Content

**FIGURE 3:**
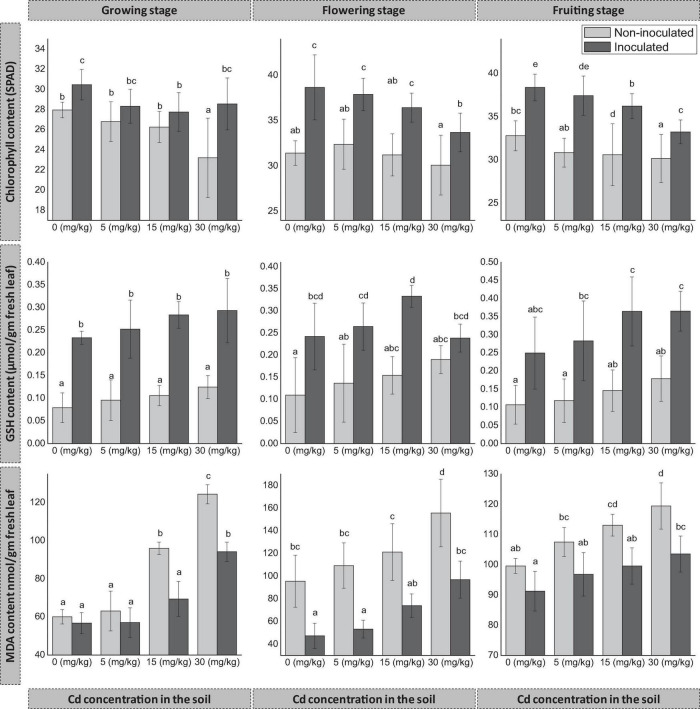
Effects of endophyte FZT214 on the chlorophyll (mean ± STD, *n* = 9), GSH, and MDA (mean ± STD, *n* = 3) content of *D. ambrosioides* grown under different Cd concentrations at different developmental stages. The different lowercase letters indicate significant variation between treatments (*p* < 0.05) according to Duncan’s test of one-way ANOVA.

The inoculation of FZT214 to *D. ambrosioides* had a positive effect on the total chlorophyll content of host plants at all three developmental stages (Figure 3). During the growing stage, the total chlorophyll content of E+ plants was more than that of E− plants, and the difference was significant (*p* < 0.05) at 0 and 30 mg kg^–1^. During the flowering and fruiting stages, the total chlorophyll content of E+ plants was significantly (*p* < 0.05) more than that of E− plants at all concentrations of Cd. At the same time, with the increase in soil Cd concentrations, the total chlorophyll content was decreased for both E+ and E− plants in most of the cases, except at 30 mg kg^–1^ for E+ plants during the growing stage.

#### Glutathione and Malondialdehyde

The inoculation of FZT214 to *D. ambrosioides* affected host plants’ GSH and MDA content (Figure 3). The GSH content of E+ plants was more than that of E− plants. The difference was significant (*p* < 0.05) at most conditions during different developmental stages, except at 30 mg kg^–1^ during the flowering stage (*p* > 0.05) and 0 mg kg^–1^ during the fruiting stage (*p* > 0.05). The increase in soil Cd concentrations supported the increase of GSH content in both E+ and E− plants at most of the cases except at 30 mg kg^–1^ for E+ plants during the flowering stage. The MDA content of E+ plants was less than that of E− plants. The difference was significant (*p* < 0.05) at most conditions during different developmental stages, except at 0 and 5 mg kg^–1^ during the growing and fruiting stages (*p* > 0.05).

### Effects of FZT214 on the Phytohormone Content of *D. ambrosioides*

**FIGURE 4:**
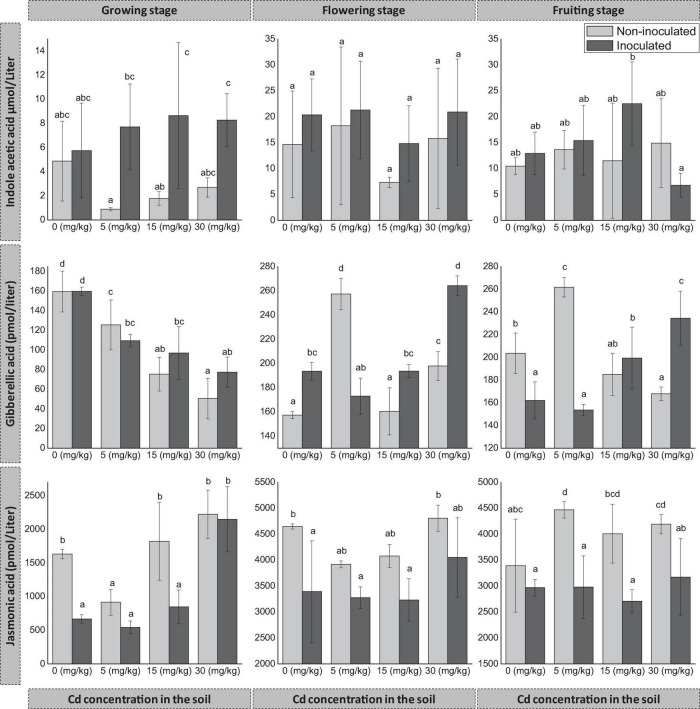
Effects of endophyte FZT214 on the endogenous phytohormone content of *D. ambrosioides* grown under different Cd concentrations at different developmental stages. The different lowercase letters indicate significant variation between treatments (*p* < 0.05) according to Duncan’s test of one-way ANOVA (mean ± STD, n = 3).

The inoculation of FZT214 to *D. ambrosioides* affected host plants’ phytohormone content. However, the change was variable with Cd concentration in the soil and developmental stages (Figure 4). The inoculation increased host plants’ IAA content except at 30 mg kg^–1^ Cd at the fruiting stage. However, the difference was only significant (*p* < 0.05) at 5 and 15 mg kg^–1^ Cd during the growing stage (Figure 4). GA content of E+ plants was more than that of E- plants, except at 5 mg kg^–1^ Cd during all developmental stages and 0 mg kg^–1^ Cd during the growing stage. The difference was significant (*p* < 0.05) during the flowering stage at all Cd concentrations, while the difference was non-significant (*p* > 0.05) during the growing stage at all Cd concentrations. During the fruiting stage, the difference was significant (*p* < 0.05) at all Cd concentrations except at 15 mg kg^–1^ Cd. On the contrary, JA content of E+ plants was comparatively less than that of E− plants during all developmental stages (Figure 4). During the growing stage, the difference was significant (*p* < 0.05) at 0 and 15 mg kg^–1^. During the flowering stage, the difference was only significant (*p* < 0.05) at 0 mg kg^–1^. During the fruiting stage, the difference was significant (*p* < 0.05) under various Cd exposures except (*p* > 0.05) at 0 mg kg^–1^ Cd.

## Discussion

### Growth Improvement

Previous studies have demonstrated that endophytes can enhance host plant growth under Cd stress (Soleimani et al., 2010; Khan et al., 2015; Zhu et al., 2018; He et al., 2019; Shahid et al., 2019; Zhang et al., 2019). In the present study, it was found that fungal endophyte FZT214 improved *D. ambrosioides* growth under Cd stress. The dry biomass of both aboveground and belowground parts of E+ plants was more than that of E− plants. However, the difference depends on the developmental stage of plants and the concentration of Cd in the soil. At the flowering and fruiting stages, the shoot length and dry biomass of aboveground parts were significantly (*p* < 0.05) improved, while at the growing stage, there was an increase but not significant (*p* > 0.05). At the growing and flowering stage, the root length of E+ plants was longer than that of E− plants at all Cd concentrations except at 5 mg kg^–1^ in the growing stage. This trend was opposite during the fruiting stage, except at 30 mg kg^–1^ in the soil (Figure 1). Understanding this phenomenon (less biomass and more length) needs more work. Overall, E+ plants had shown positive developmental effects than E− plants under various Cd stress levels. However, signs of Cd toxicity were observed after 90 days on the leaves of plants grown in 15 and 30 mg kg^–1^ Cd in the soil, regardless of inoculation; these plants exhibited some yellow spots especially on the old leaves’ surface. The reason for this phenomenon could be various physiological changes like a surge of abscisic acid or ethylene as a result of the Cd stress (Zhou and Qiu, 2005).

Seed endophytes have been associated with enhanced plant growth–related properties such as nutrient uptake, reduced susceptibility to heat and drought stress, and improved seed production (Hubbard et al., 2012, 2014; Li et al., 2019). This study indicated a comparative reduction in the seed dry biomass with the increase in the soil Cd both in E+ and E− plants except at 15 mg kg^–1^ Cd for E+ plants (Figure 2). This can be due to the effect of Cd stress. An important fact to be noted is that the dry seed biomass of E+ plants was significantly (*p* < 0.05) higher than that of E− plants. Indeed, we also observed early flowering in E+ plants as compared with E+ plants. Similarly, Gao and Shi (2018) reported successful transmission of seed endophyte *Herbaspirillum frisingense* RE3-3 to the seedlings, which improved seedling development and growth under Cd stress. These results suggest the possible survival strategy of plants under Cd stress conditions where the symbiotic association with the seed endophyte provides growth and tolerance benefits as well as helps the host plant to produce enough seeds for the next generation.

### Cd Accumulation and Translocation

As described previously, *D. ambrosioides* is a Pb hyperaccumulator that can be used for phytoextraction and/or phytostabilization. For the effective and successful phytoremediation process, it is of utmost importance that the plant used could be able to withstand high metal stress. In addition, constant metal absorption and translocation to the aboveground parts is also very crucial. In general, the Cd concentration was relatively less in the aboveground and belowground tissues of E+ plants in most of the treatments (Table 1). This finding was similar to the results of the previous studies that observed lower Cd in inoculated plants than non-inoculated plants (Wang et al., 2016; He et al., 2017; Zhan et al., 2017; Zhu et al., 2018; Shahid et al., 2019). However, in this study, the difference was non-significant (*p* > 0.05) in most of the tested conditions (Table 1).

Khan et al. (2015) recorded lower Cd in the leaf and roots of *Serratia* sp.–inoculated *Solanum nigrum* grown under Cd stress, and higher Cd in the stems of the inoculated plants. Wang et al. (2016) also observed that the dark septate endophyte colonization to *Zea mays* reduced Cd in the shoots and roots under Cd stress. He et al. (2017) reported that overall Cd content was less in the shoots and roots of inoculated plants in most of the treatments. However, the total Cd accumulated per plant was higher in the inoculated plants due to their higher biomass. This “dilution mechanism” can be related to the lower Cd toxicity in plant tissue and subsequently increased plant biomass in the inoculated plants (Guo et al., 2013). The induced growth and tolerance by the inoculated endophyte can be due to the relative decrease in metal uptake into the above and belowground tissues and the selective translocation of Cd from roots to aboveground tissues (Zhu et al., 2018; Shahid et al., 2019). Generally, metal contents in plant samples are subject to the availability of metals in the soil (Vivas et al., 2006). Other possible reasons for lower Cd in the inoculated plants could be Cd sequestration in the root zone, siderophore secretion and production of exopolysaccharides, excretion of Cd from the cell by efflux pumps, and reduced phytotoxicity through secretion of antioxidants (Shahid et al., 2019; Wang et al., 2019).

The metal accumulation by a plant depends on the availability of the metal in the root zone of the plant (Hinsinger et al., 2009); therefore, the rhizospheric soil of E+ and E− plants was tested for the Cd concentration after the harvest. The metal content and its bioavailability in the rhizospheric zone of the plant are often considered as the better indicator of the metal translocation to the plant than the total soil (Cheng et al., 2018; Campillo-Cora et al., 2019). In general, the total and bioavailable Cd in the rhizospheric soil of E+ plants was less than that of E− plants, but the difference was non-significant (*p* > 0.05), except for some conditions (Table 2). Besides, the difference between E+ and E− plants was inconsistent with the Cd concentration in the soil and developmental stage of host plants. In addition, the belowground tissues accumulated more Cd than the aboveground tissues; it indicates that only little Cd was translocated to the aboveground parts. Khan et al. (2017) reported greater Cd contents in the *Solanum nigrum* roots, which suggest the role of these plants as a phytostabilizer. More Cd accumulation in the belowground tissue also indicates that the roots are the major site for storage of metals (Shahid et al., 2019).

Interestingly, the Cd concentration in the aboveground parts was more during the growing stage than the latter stages, irrespective of the soil Cd concentration and the endophyte inoculation. It indicates that the Cd accumulation and translocation were relatively higher during the growing stage than the latter stages. The BAF and TF were comparatively higher at the growing stage (Table 3). Therefore, the impact of Cd was greater while the plants were young; this could be the possible reason that the difference between shoot and root dry biomass of inoculated and non-inoculated plants was lower (*p* > 0.05) at the growing stage while higher (*p* < 0.05) at the latter stages (Figure 1).

### Physiological and Biochemical Changes

The chlorophyll content is a significant indicator of plant growth status (Chen et al., 2010). Moreover, Cd-induced toxicity in plants can negatively influence the biosynthesis of chlorophyll by preventing protochlorophyllide reductase activity required for chlorophyll synthesis and altering the photosynthetic electron transport at PS-II (Somashekaraiah et al., 1992; Neelu, Kumar et al., 2000). In the present study, the observed decrease in the chlorophyll content with the increase of Cd concentration in the soil was probably due to Cd stress (Figure 3); a similar trend was also reported by several previous researchers (Zhang et al., 2010; Kamran et al., 2015). Our finding of relatively higher chlorophyll content in E+ plants agrees with the finding of Hunt et al. (2005) that endophytic-inoculated perennial ryegrass plants had higher chlorophyll content.

High exposure of Cd to the plants induces Cd stress that, in turn, increases ROS in the plant tissues. ROS in the form of superoxide anion and hydrogen peroxide mimic and disrupt the regular cellular functions by shifting the oxidation/reduction cycle (Khan et al., 2015). Glutathione is one of the important ROS scavenging molecules in plants. The free glutathione exists chiefly in its reduced form (GSH), its relatively higher production in the stress-adapted plants is due to strong activation of the defense cycle (Tausz et al., 2004). In this study, GSH content of E+ plants was more than that of E− plants (Figure 3), which indicates the counteractive mechanism developed by inoculated endophyte to check oxidative stress due to Cd. The results were consistent with previous studies, which observed endophytic inoculation may improve the host plant growth and tolerance to Cd stress through GSH regulation in different host plants (Khan et al., 2015; Wang et al., 2016; He et al., 2017; Zhan et al., 2017; Zhang et al., 2019).

Lipid peroxidation is an indicator of oxidative damage of the plant under metal stress; primarily it disturbs functions and integrity of cell membrane, and the damage is often irreversible (Khan and Lee, 2013; Khan et al., 2015; Bilal et al., 2018). Malondialdehyde (MDA) is a secondary breakdown product of lipid peroxides; its low levels imply a lesser degree of lipid peroxidation. The relatively lower MDA in E+ plants is due to lower lipid peroxidation, which suggests that the endophyte FZT214 interacts with the host plant and has some synergistic role in the protection of the host from Cd stress. The results were consistent with previous studies that lower MDA contents were found in endophyte-infected plants (Khan and Lee, 2013; Khan et al., 2015; Wang et al., 2016; Bilal et al., 2018; Zhu et al., 2018). The increase in soil Cd concentrations showed some signs of Cd toxicity in terms of an increase in the MDA content for both E+ and E− plants, but still E+ plants were more tolerant with lesser MDA content.

Previous studies have shown that some endophytic fungi can exogenously produce phytohormones that can help the host plant to mitigate the effects of abiotic stress (Khan et al., 2012). In liquid culture, the secondary metabolites of FZT214 showed the presence of IAA, GA, and JA (Supplementary Table 1). Interestingly, FZT214 inoculation increased IAA content of E+ plants as compared with E− plants (Figure 4). IAA has been reported to play a key role in apical dominance, cell elongation, and development of vascular tissue (Wang et al., 2001). It can also modulate plant growth under stress conditions (Eyidogan et al., 2012). The relatively higher endogenous IAA production in E+ plants than E− plants in the current study may be related to the increased shoot length and biomass in the inoculated plants (Figures 1, 4).

GA is an important phytohormone that plays a critical role in plant development such as stem elongation, flower and trichome initiation, seed germination, fruit development, and leaf expansion (Yamaguchi, 2008; Liu et al., 2009). JA had also been demonstrated to be able to promote plant performance under unfavorable conditions such as metal stress (Bilal et al., 2018; Per et al., 2018). The relatively higher endogenous GA production in E+ plants than E− plants especially at 15 and 30 mg kg^–1^ Cd in the current study indicates better plant growth and can also be related to the increased seed production (Figure 2). JA was reported to alter antioxidant potential and reducing H_2_O_2_, MDA concentrations, and additionally improve photosynthetic pigment concentrations under Pb and Cd stress in different plants (Piotrowska et al., 2009; Ahmad et al., 2017). JA is related to plant response to Cu and Cd toxicity with differential effects amidst the plant and growth stage (Maksymiec et al., 2005). It induces the production of defensive proteins called jasmonate inducible proteins and other metabolic changes (Farmer et al., 2003; Maksymiec et al., 2005). These changes have been related to several developmental processes of plants; for example, it may reduce photosynthetic activity and growth processes, and commence harmful effects (Maslenkova et al., 1990; Maciejewska and Kopcewicz, 2002; Maksymiec et al., 2005).

The relatively lower JA observed in E+ than E− plants indicates better plant tolerance to Cd stress. Kim et al. (2014) also observed reduced contents of endogenous JA in rice under Cd stress. Bilal et al. (2018) observed higher GA, and lower ABA and JA in the endophyte inoculated plants under Al/Zn stress. To further test the role of FZT214 in host-plant physiology under Cd-exposed conditions, this isolate can also be assessed in the future on phytohormone-deficient mutant plant cultivars, e.g., GA-deficient mutant rice cultivar (Waito-C) and GA cultivar (Dongjin-byeo) (Khan et al., 2012). The symbiotic interaction of the plant–endophyte alleviates the abiotic stress by inducing various physiological changes like reduced glutathione, catalase, peroxidase, and polyphenol oxidase, besides endophytic interaction modulates the plant hormone levels such as downregulation of abscisic acid, change in jasmonic acid, and increase in salicylic acid contents (Waqas et al., 2012; Bilal et al., 2018).

## Conclusion and Remarks

If not all, most seed endophytes are directly transferred from their parents to progeny. Furthermore, their existence or colonization could play an essential role in the successive germination and plant growth. This study explores the role of fungal seed endophyte FZT214 to *D. ambrosioides* during its different developmental stages under various Cd stress conditions. The results showed that FZT214 colonization supported *D. ambrosioides* growth under variable Cd stress, through all three developmental stages as evident by the increased plant growth parameters, higher chlorophyll content, lower oxidative damage revealed by the lower lipid peroxidation, and higher GSH content. The beneficial effects of the FZT214 were more pronounced under Cd-spiked soil, explaining its effectiveness in decreasing the Cd toxicity in E+ plants.

The FZT214 colonization also significantly improved the seed production of *D. ambrosioides* under Cd stress conditions and induced early flowering. The results suggested that endophyte-colonized *D. ambrosioides* may have an ecological and evolutionary advantage in the metal-contaminated environment as compared with the non–endophyte-treated plants. Seed endophytic isolate FZT214 was recorded to exogenously secrete phytohormones such as IAA, GA, and JA. The phytohormone-producing characteristics of this fungal isolate might be the key mechanism of the host plant growth promotion.

Moreover, a deep understanding of the molecular mechanism of the interaction between host plant–endophytes–metal is a crucial requirement in the future, and more studies are required concerning FZT214 potential in other plants for phytoremediation and growth promotion in the Cd-contaminated environment.

## Data Availability Statement

The original contributions presented in the study are included in the article/Supplementary Material, further inquiries can be directed to the corresponding author/s.

## Author Contributions

SP, VKS, and HL hypothesized the research work. SP and VKS performed the research, analyzed the data, wrote the manuscript, and review, and edited the manuscript. TL performed the methodology. WT investigated the project and resources, project administration. HL acquired the funding and supervised the research. All authors contributed to the article and approved the submitted version.

## Conflict of Interest

The authors declare that the research was conducted in the absence of any commercial or financial relationships that could be construed as a potential conflict of interest. The handling editor declared a past co-authorship with one of the authors VKS.

## Publisher’s Note

All claims expressed in this article are solely those of the authors and do not necessarily represent those of their affiliated organizations, or those of the publisher, the editors and the reviewers. Any product that may be evaluated in this article, or claim that may be made by its manufacturer, is not guaranteed or endorsed by the publisher.

## Footnotes

1
www.mlbio.cn

## Abbreviations

ABA: abscisic acid
BAF: bioaccumulation factor
DTNB: dithionitrobenzoic acid
DTPA-TEA: diethylenetriaminepentaacetic acid–triethanolamine
E+: inoculated plants
E−: non-inoculated plants
GA: gibberellic acid
GSH: glutathione
T-GSH: total glutathione
GSSG: oxidized glutathione
HM: heavy metal
IAA: indole-3-acetic acid
JA: jasmonic acid
MDA: malondialdehyde
PTEs: potentially toxic elements
TF: translocation factor.
