# Supplementary material for: Fungal Seed Endophyte FZT214 Improves Dysphania ambrosioides Cd Tolerance Throughout Different Developmental Stages
Source: Front Microbiol. 2022 Jan 4;12:783475. doi: 10.3389/fmicb.2021.783475 (PMC8764135; doi:10.3389/fmicb.2021.783475)
Supplement: Supplementary file 1 [file Table_1.docx]

**Table 1 Concentration of the different phytohormones (Mean ± STD, n = 3) in the crude metabolites of the FZT214**

|  | **Concentration** |
| --- | --- |
| **Jasmonic acid** | 184.46±23.10 pM L^-1^ |
| **Gibberellic acid** | 13.38±1.01 pM L^-1^ |
| **Indole acetic acid** | 2.36±0.34 µM L^-1^ |
